# Supplementary material for: In Silico Neuroprotective Effects of Specific Rheum palmatum Metabolites on Parkinson’s Disease Targets
Source: Int J Mol Sci. 2023 Sep 11;24(18):13929. doi: 10.3390/ijms241813929 (PMC10530814; doi:10.3390/ijms241813929)
Supplement: Supplementary file 1 [file ijms-24-13929-s001.zip › ijms-2535003-supplementary.pdf]

Supplementary Material for "In Silico Neuroprotective Effects of Specific *Rheum palmatum* Metabolites on Parkinson's Disease Targets"

Table S1. List of *R. palmatum* major metabolites and generated ligand forms.

Ligand	Exact Molecular Weight	Ionization pH	Structure
Aloe-emodin (CID: 10207)			
Form 1	254.058	6.84	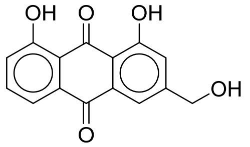
Form 2	254.058	6.84	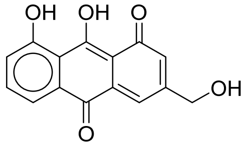
Form 3	254.058	6.84	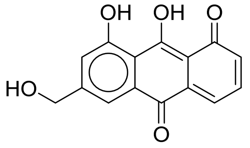
Form 4	253.05	6.84	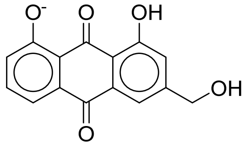
Form 5	253.05	6.84	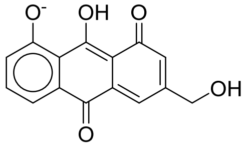
Form 6	252.042	7.17	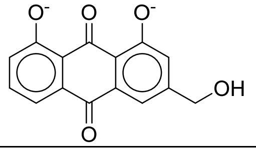
Chrysophanol (CID: 10208)			
Form 1	254.058	6.84	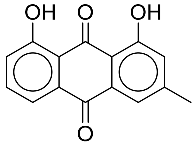
Form 2	254.058	6.84	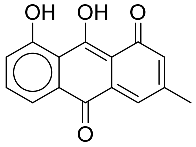

Form 3	254.058	6.84	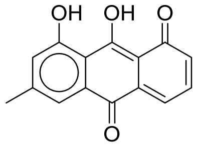
Form 4	253.05	6.84	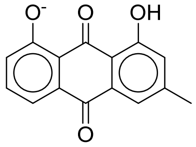
Form 5	253.05	6.84	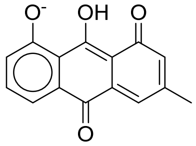
Form 6	252.042	7.17	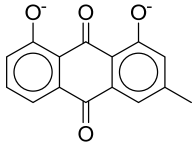
Emodin (CID: 3220)			
Form 1	269.045	7.17	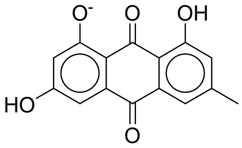
Form 2	269.045	7.17	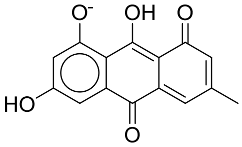
Form 3	269.045	7.17	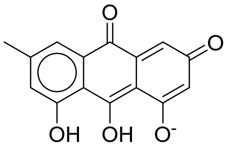
Form 4	268.037	7.17	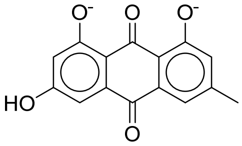
Form 5	268.037	7.17	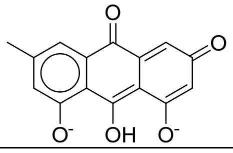
Physcion (CID: 10639)			
Form 1	284.068	6.91	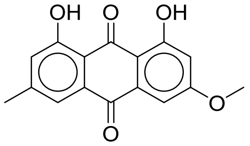

Form 2	284.068	6.91	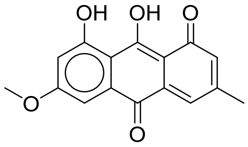
Form 3	284.068	6.91	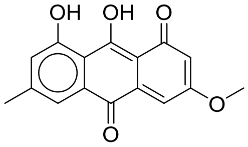
Form 4	283.061	6.91	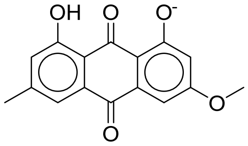
Form 5	283.061	6.91	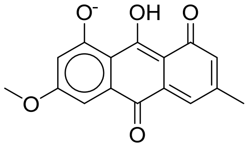
Form 6	282.053	7.17	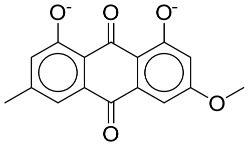
Rhein (CID: 10168)			
Form 1	283.024	6.93	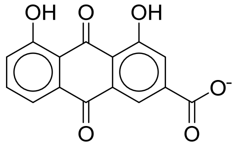
Form 2	283.024	6.93	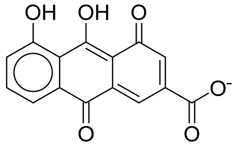
Form 3	283.024	6.93	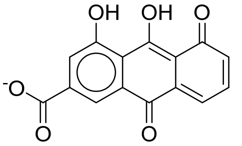
Form 4	282.016	6.93	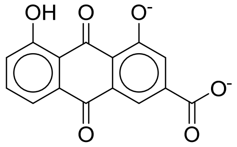
Form 5	282.016	6.93	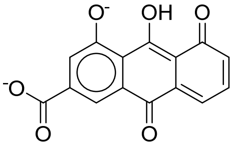
Form 6	281.009	7.7	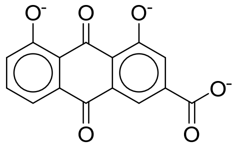

Dopamine	154.087	–	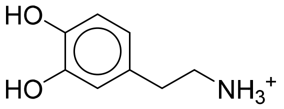
Levodopa	197.069	–	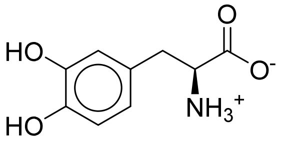

Table S2. Parkinson's disease target proteins and binding sites.

Protein	PDB ID	RMSD (Å)	Binding spheres (x y z r)	Reference
ASN	1XQ8	1.0192	235.761238 87.098921 -11.467784 12.834573	[1,2]
MAOB	2C65	0.6723	**AS1**: 56.169204 140.800776 16.387916 13.400000	PDB site records [3,4]
			**AS2**: 50.646075 154.441830 28.109744 8.900000	
			**AS3**: 28.564668 124.319410 9.517245 13.200000	
			**AS4**: 16.683806 129.076451 23.305674 8.500000	
COMT	3BWM	0.6887	**AS1**: -3.065429 -9.775358 -12.231862 5.000000	PDB site records [5]
			**AS2**: 9.521219 -3.947800 -3.317792 6.000000	
			**AS3**: -8.323370 -4.020339 -16.877004 10.400000	
			**AS4**: -4.031587 -11.005142 -16.061175 9.100000	
$A_{2A}AR$	3EML	0.7421	**ASA1**: -8.619491 -4.022831 53.851527 9.700000	PDB site records [6]
			**ASA2**: -5.753475 -21.755505 44.032696 5.000000	
			**ASA3**: -3.104069 -15.067314 28.427817 5.000000	
			**ASA4**: -6.152340 -20.745973 46.934776 5.100000	
			**ASA5**: -1.261143 -18.640946 55.134056 5.000000	
			**ASA6**: -15.100131 -17.313670 26.230558 5.000000	
			**ASA7**: -6.194983 15.030722 8.292277 15.300000	
			**ASA8**: -19.087429 -14.862891 18.906995 5.500000	
			**ASA9**: -2.195901 11.933315 16.502750 7.100000	
			**ASB1**: 5.283943 -17.175302 60.435534 5.000000	
			**ASB2**: -4.298667 4.042169 -1.331465 5.600000	
			**ASB3**: -10.197447 12.413895 -15.647710 5.700000	
			**ASB4**: 3.681484 28.326672 -7.840765 5.000000	

AS = active site

Table S3. 3D QSAR dataset of known ASN inhibitors.

Ligand No.	Canonical SMILES	IC_{50} (nM)
Training Set		
1	C\C(=C/CO)\C=C\C=C(/C)\C=C\C1=C(C)CCCC1(C)C	190
2	COc1cc(\C=C\C(=O)[CH-]C(=O)\C=C\c2ccc(O)c(OC)c2)ccc1O	220
3	[O-][N+](=O)c1ccc2Nc3ccc(Br)cc3Sc2c1	313.8
4	[O-][N+](=O)c1ccc2Nc3ccc(I)cc3Sc2c1	441.5
5	[O-][N+](=O)c1ccc2Nc3ccccc3Sc2c1	485
6	COc1ccc2Nc3ccc(OC)cc3Sc2c1	507.1
7	O[C@@H]1Cc2c(O)cc(O)cc2O[C@@H]1c3ccc(O)c(O)c3	800
8	Oc1cc(cc(O)c1O)C(=O)Nc2ccc(NC(=O)c3ccc([O-])cc3)cc2	980
9	[O-][N+](=O)c1ccc2[nH]c(\C=C\C=C\c3ccccn3)nc2c1	1,090
10	Nc1ccc(NC(=O)c2cc(O)c(O)c(O)c2)cc1	1,340
11	COc1ccc2Nc3ccc(cc3Sc2c1)C#N	1,440
12	o1c(\C=C\C=C\c2ccncc2)nc3ccccc13	1,850
13	Clc1ccc2nc(oc2c1)\C=C\C=C\c3ccncc3	2,190

14	C\C(=C/C=C/C(=C/C(=O)[O-])/C)\C=C\C1=C(C)CCCC1(C)C	3,000
15	CNc1ccc2[N@H+](C)c3ccc(OC)cc3Sc2c1	3,329
16	COc1ccc2N(C)c3ccc(N)cc3Sc2c1	3,928
17	Oc1cc(cc(O)c1[O-])C(=O)[O-]	4,430
18	Cc1ccc2oc(\C=C\C=C\c3ccncc3)nc2c1	4,430
19	Oc1cc(O)c2cc(O)c([o+]c2c1)c3cc(O)c(O)c(O)c3	6,500
20	CCC[N@@H+]1C[C@H](CSC)C[C@H]2[C@H]1Cc3c[nH]c4cccc2c34	7,900
21	Oc1c([O-])cc2OC(=CC(=O)c2c1O)c3ccccc3	8,200
22	O[C@H]1Cc2c(O)cc(O)cc2O[C@@H]1c3cc(O)c(O)c(O)c3	8,900
23	Oc1cc(O)c2cc(O)c([o+]c2c1)c3ccc(O)c(O)c3	10,300
24	O[C@@H]1Cc2c(O)cc(O)cc2O[C@@H]1c3cc(O)c(O)c(O)c3	10,600
25	COc1ccc2[N@@H+](C)c3ccc(cc3Sc2c1)N(C)C	10,736
26	OC1=CC=Cc2cc(O)c(O)c(O)c2C1=O	12,900
27	CC1=CC(=O)c2ccccc2C1=O	15,000
28	O=C1C=CC(=O)c2ccccc12	15,000
29	Oc1cc(ccc1[O-])C2=C([O-])C(=O)c3c(O)cc([O-])cc3O2	20,000
30	CC(=O)N1c2ccccc2Sc3cc(ccc13)[N+](=O)[O-]	23,491
31	Oc1cc(ccc1[O-])C2=CC(=O)c3c(O)cc([O-])cc3O2	28,000
32	CC(=O)N1c2ccc(O)cc2Sc3cc(ccc13)[N+](=O)[O-]	32,074
33	C[C@]12CC[C@H]3[C@@H](CCC4=CC(=O)CC[C@]34C)[C@@H]1CCC2=O	85,900
34	C[C@]12CC[C@H]3[C@@H](CCc4cc(O)ccc34)[C@@H]1CCC2=O	93,100
35	C[C@]12CC[C@H]3[C@@H](CCC4=CC(=O)CC[C@]34C)[C@@H]1CC[C@@H]2O	96,800
36	COc1cc(\C=C\c2cc(\C=C\c3ccc(O)c(OC)c3)[nH][nH+]2)ccc1O	126,800
Test Set		
1	Oc1ccc(C[C@@H](OC(=O)\C=C\c2ccc(O)c(O)c2)C(=O)[O-])cc1O	210
2	C[C@H](Cc1ccc(O)c(O)c1)[C@H](C)Cc2ccc(O)c(O)c2	210
3	C[C@H](Cc1ccccc1)[N@H+](C)CC#C	270
4	[NH3+]CCc1ccc(O)c(O)c1	530
5	Oc1cc(cc(O)c1O)C(=O)Nc2ccc(NC(=O)c3ccc(F)cc3)cc2	1,700
6	Clc1ccc(NC(=O)c2ccc(cc2)C(=O)\C=C\C=C\c3ccncc3)cc1	2,050
7	COc1ccc2N(C(=O)C)c3ccc(cc3Sc2c1)[N+](=O)[O-]	14,031
8	C[NH+]1CCN(CC1)[NH2+]CC[NH+]2c3ccccc3Sc4ccccc24	16,000
9	C[C@]12CC[C@H]3[C@@H](CCc4cc(O)ccc34)[C@@H]1CC[C@@H]2O	60,000

Table S4. 3D QSAR dataset of known MAOB inhibitors.

Ligand No.	Canonical SMILES	IC_{50} (nM)
Training Set		
1	C[C@H](Cc1ccccc1)[N@H+](C)CC#C	19
2	FC(F)(F)c1ccc(\C=C\C2CC[NH+](CC#C)CC2)cc1	18.6
3	CN(CC#C)CC(=C)c1ccc(Cl)cc1	120
4	CN(CC#C)CC(=C)c1ccc(F)c(C)c1	230
5	CN(CC#C)[C@H](CF)CC(=C)C	664
6	Fc1cc(F)c(\C=C\C2CC[NH+](CC#C)CC2)cc1F	436.2
7	Fc1ccc(\C=C\[C@@H]2CCC[N@H+](CC#C)C2)cc1	1,469.50
8	FC(F)(F)c1ccc(\C=C/C2CC[NH+](CC#C)CC2)cc1	1,506.60
9	CC(C)c1ccc(\C=C/C2CC[NH+](CC#C)CC2)cc1	1,883.90
10	Fc1ccccc1\C=C\C2CC[NH+](CC#C)CC2	11,830.40
11	Fc1ccccc1CCC2CCN(CC#C)CC2	14,159.50
12	Cc1ccc(\C=C/C2CC[NH+](CC#C)CC2)cc1	19,162.80
13	Fc1cccc(\C=C/C2CC[NH+](CC#C)CC2)c1	48,912
14	[NH3+][C@H]1C[C@H]1c2ccccc2	33

15	COc1cccc(c1)C(=C)CN(C)CC#C	200
16	[NH3+]C[C@@]1(C[C@@H]1F)c2ccccc2	12
17	Cc1cccc(COc2cccc(Br)c2)c1	1,980
18	Brc1cccc(OCc2ccccc2)c1	2,730
19	CN1N=C(C[C@@H]1c2cccc(F)c2)c3ccccc3	60
20	Cc1ccc2oc(cc2c1)c3ccccc3Br	200
21	CN1N=C(C[C@H]1c2ccccc2C)c3ccccc3	110
22	Cc1ccc(cc1)C2=Cc3cc(C)ccc3OC2=O	0.3085
23	CN1N=C(C[C@@H]1c2ccc(F)cc2)c3ccccc3	140
24	CCC(C)(C)c1ccc(OCCC[N@@H+]2CCC[C@@H](C)C2)cc1	428
25	CN(CC#C)CC(=C)c1ccc(COc2ccccc2)cc1	620
26	CC[C@@]1([NH3+])C[C@@H]1c2ccccc2	190
27	CN1N=C(C[C@@H]1c2ccccc2F)c3ccccc3	280
28	Brc1ccccc1c2oc3ccccc3c2	1,130
29	Brc1cccc(c1)c2oc3ccccc3c2	1,170
30	COc1ccc2C(=O)\C(=C\c3ccccc3)\Cc2c1	9.2
31	CC(=O)c1ccc(OCc2ccc(F)cc2)cc1	12.9
32	Cc1ccc(cc1)C2=CC(=S)c3ccc(F)cc3O2	1,610
33	CCC(C)(C)c1ccc(OCCC[NH+]2CCC(C)CC2)cc1	2,070
34	CC(=O)c1cccc(OCc2ccc(Cl)cc2)c1	10.52
35	Cc1ccc(COc2ccc3C(=O)CCCc3c2)cc1	11
36	CCC[C@@]1([NH3+])C[C@H]1c2ccccc2	600
37	CC(=O)c1ccc(OCc2cccc(Cl)c2)cc1	21.5
38	Fc1cccc(COc2ccc3C(=O)CCCc3c2)c1	17
39	Cc1cccc2cc(oc12)c3ccccc3	10,730
40	CC(=O)c1ccc(OCc2ccc(F)cc2F)cc1	27.3
41	Fc1ccc(\C=C\C2=COc3ccccc3C2=O)cc1	8.6
42	CC(=O)c1ccc(OCc2ccccc2F)cc1	56.9
43	COc1ccc2OC(=O)C(=Cc2c1)c3ccc(C)cc3	1.52
44	Clc1cccc(COc2ccc3C(=O)CCCc3c2)c1	15
45	CN(CC#C)CC(=C)c1cccc(CNc2ccccc2)c1	1,400
46	Clc1ccc(COc2ccc3COC(=O)c3c2)cc1	2.8
47	Fc1ccc(COc2ccc3COC(=O)c3c2)cc1	6.4
48	Clc1ccc(CN[C@H]2C[C@@H]2c3ccccc3)cc1	14,100
49	CC(=O)c1ccc(OCc2cccc(C)c2)cc1	86.3
50	COc1ccc(cc1)C(=O)\C=C\c2ccccc2F	56
51	CC(=O)c1cccc(OCc2ccc(F)cc2)c1	86.4
52	Cc1ccc2C(=O)C[C@H](Oc2c1)c3ccccc3	110
53	CC(=O)c1ccc(OCc2ccc(C)cc2)cc1	102
54	Cc1ccc2CC(=O)C(=Cc2c1)C(=O)[O-]	19.05
55	CC(=O)c1cccc(OCc2cccc(c2)C(F)(F)F)c1	21.2
56	CC1=C(C)c2ccc(OCc3cccc(F)c3)cc2OC1=O	0.912
57	CC(=O)c1ccc(OCc2ccc(Cl)cc2)cc1	63.2
58	Cc1ccc2C(=O)C[C@@H](Oc2c1)c3ccccc3	140
59	CC(=O)c1cccc(OCc2ccc(C)cc2)c1	140
60	COc1ccc(cc1)C(=O)\C=C\c2ccc(Cl)cc2	54
61	CN(CC#C)Cc1ccc(O)c2ncccc12	57
62	Clc1ccc(COc2ccc3OC=CC(=O)c3c2)cc1	2
63	Fc1ccc(COc2ccc3C(=O)CCOc3c2)cc1	8.6
64	Cc1ccc(COc2ccc3OC=CC(=O)c3c2)cc1	6
65	Cc1ccc(COc2ccc3SC(=O)Oc3c2)cc1	5
66	CN1N=C(C[C@H]1c2ccccc2Cl)c3ccccc3	3,650
67	Clc1cccc(COc2ccc3OCCOc3c2)c1	57

68	CC(=O)c1cccc(OCc2ccc(cc2)C(F)(F)F)c1	43.2
69	Fc1cccc(COc2cccc3[C@@H](CCc23)NCC#C)c1	280
70	Cc1ccc(cc1)[C@H]2CC(=O)c3ccc(F)cc3O2	150
71	Cc1ccc(cc1)[C@@H]2CC(=O)c3ccc(F)cc3O2	150
72	Fc1ccc(cc1)c2csc(NN=C3CCCCC3)n2	3.802
73	[NH3+][C@H]1C[C@]1(F)c2ccc(F)cc2	4,900
74	Cc1cccc2cc(oc12)c3ccccc3Br	9,680
75	Clc1cccc(COc2ccc3OC=CC(=O)c3c2)c1	3.6
76	Cc1ccc(cc1)[C@H]2CC(=O)c3ccc(F)cc3O2	160
77	Fc1ccc(cc1)[C@H]2CC(=O)c3ccc(F)cc3O2	150
78	CC1=C(Cl)C(=O)Oc2cc(OCc3ccccc3)ccc12	2.1
79	Clc1ccc(COc2ccc3C(=O)OCc3c2)cc1	13.87
80	Fc1ccc(COc2ccc3OC=CC(=O)c3c2)cc1	8.4
81	Fc1ccc(cc1)[C@@H]2CC(=O)c3ccc(F)cc3O2	170
82	COc1ccc2C(=O)C(=COc2c1)\C=C\c3ccc(F)cc3	3.1
83	Cc1ccc(cc1)[C@@]2(F)C[C@H]2[NH3+]	6,700
84	Oc1ccc2C(=O)\C(=C\c3ccc(Cl)cc3)\Cc2c1	13
85	Clc1ccc(\C=C\2/COc3ccccc3C2=O)cc1	154.23
86	CC(=O)c1ccc(OCc2ccc(cc2)C(F)(F)F)cc1	77.8
87	COc1ccc(cc1)C2=Cc3cc(C)ccc3OC2=O	13.05
88	CN(C)c1ccc(\C=C\2/Oc3ccccc3C2=O)cc1	99
89	[NH3+][C@H]1C[C@]1(F)c2ccc(Cl)cc2	3,700
90	Fc1cc(NC(=O)c2ccc3[nH]ncc3c2)ccc1Cl	0.6607
91	O=C1CCOc2cc(OCc3ccccc3)ccc12	57.37
92	Cc1cccc(COc2ccc3OC=CC(=O)c3c2)c1	19
93	Cc1ccc(NC(=O)C2=COc3ccccc3C2=O)cc1C	0.67
94	CC(=O)c1ccc(OCc2ccccc2Cl)cc1	311
95	Fc1ccc(COc2cccc(C=O)c2)cc1	930
96	Fc1ccc2C(=O)C[C@@H](Oc2c1)c3ccccc3	650
97	COc1ccc2C(=O)C(=COc2c1)\C=C\c3ccccc3	16
98	Brc1ccc(COc2cccc(C=O)c2)cc1	190
99	Fc1ccc(COc2ccc3C(=O)C=COc3c2)cc1	25
100	Cc1ccc(cc1)[C@@]2(F)C[C@@H]2[NH3+]	13,000
101	COc1ccc(\C=C\2/COc3ccccc3C2=O)cc1	58.9
102	O=C1C=COc2ccc(OCc3ccccc3)cc12	53
103	Cc1ccc(cc1)[C@@H]2CC(=O)c3ccc(C)cc3O2	500
104	Cc1ccc(cc1)[C@H]2CC(=O)c3ccc(C)cc3O2	530
105	CC(=O)c1ccc(OCc2ccccc2C(F)(F)F)cc1	183
106	Clc1cccc(COc2ccc3C(=O)C=COc3c2)c1	17
107	O=C1Oc2cc(COc3ccccc3)ccc2C=C1	64.57
108	Clc1ccc(COc2ccc3C(=O)CCOc3c2)cc1	33.45
109	Oc1ccc2C(=O)\C(=C\c3cccc(F)c3)\Cc2c1	84
110	Cc1ccc(cc1)[C@@H]2CC(=O)c3ccc(C)cc3O2	590
111	Fc1ccc(CCOc2ccc3COC(=O)c3c2)cc1	68
112	CC(=O)c1cccc(OCc2cccc(C)c2)c1	893
Test Set		
1	CN(CC#C)Cc1ccccc1	8.2
2	CN(CC#C)CC(=C)c1ccc(Cl)c(C)c1	100
3	Cc1ccc(\C=C\C2CC[NH+](CC#C)CC2)cc1	147
4	C#CC[NH+]1CCC(CC1)\C=C/c2ccc(cc2)C3CC3	2,640.10
5	FC(F)(F)c1cccc(\C=C/C2CC[NH+](CC#C)CC2)c1	9,352.50
6	F[C@H]1Cc2ccccc2[C@@H]1[NH2+]CC#C	27
7	CCC(C)(C)c1ccc(OCCC[N@H+]2CCCC[C@@H]2C)cc1	20

8	Cc1ccc(COc2cccc(Br)c2)cc1	1,530
9	Cn1ncc2cc(\C=N\c3ccc(Cl)c(Cl)c3)ccc12	1.023
10	[NH3+]C\C=C(\F)/COc1cccc(Cl)c1	30
11	Cc1cccc2cc(oc12)c3cccc(Br)c3	3,980
12	[O-]C(=O)C1=Cc2ccccc2OC1=O	17.38
13	Fc1cc(ccc1Cl)\N=C\c2ccc3[nH]ncc3c2	1.905
14	CC(=O)c1ccc(OCc2cccc(F)c2)cc1O	2.8
15	CC1=CC(=O)Oc2cc(OCCCC#C)ccc12	18.2
16	O=C1CCc2ccc(OCCCCC#C)cc2N1	52.6
17	Fc1ccc(NC(=O)c2ccc3[nH]ncc3c2)cc1Cl	0.6761
18	CC(=O)c1ccc(OCc2ccc(Br)cc2)cc1	85.6
19	C[C@@H]1CCC/C/1=N\Nc2nc(cs2)c3ccc(F)cc3	9.13
20	COc1ccc2OC(=O)C(=Cc2c1)c3cccc(C)c3	17.05
21	Oc1ccc(cc1)C(=O)\C=C\c2ccc(Cl)cc2	31
22	COc1ccc2C(=O)\C(=C\c3ccc(cc3)N(C)C)\Cc2c1	60
23	[NH3+][C@@H]1C[C@]1(F)c2ccc(Cl)cc2	4,800
24	Fc1ccc(cc1F)c2cc3C(=O)c4ccccc4c3nn2	5.012
25	Fc1ccc2C(=O)C[C@H](Oc2c1)c3ccccc3	620
26	[NH3+]C\C=C(\F)/COc1ccc(cc1)C(F)(F)F	130
27	Fc1cccc(COc2cccc(C=O)c2)c1	1,210
28	[NH3+]CCc1ccc(OCc2cccc(F)c2)cc1	193

Table S5. 3D QSAR dataset of known COMT inhibitors.

Ligand No.	Canonical SMILES	IC_{50} (nM)
Training Set		
1	[O-]c1c(F)cc(c2cccnc12)S(=O)(=O)C3CCCC3	1.995
2	[O-]c1c(F)cc(c2cccnc12)S(=O)(=O)N3CCCC3	3.981
3	Cc1cccc(C)c1CN2CCN3C=C([O-])C(=O)C=C3C2	6.3
4	Oc1ccc(c2cccnc12)S(=O)(=O)C3CCCC3	6.31
5	Cc1ccc(cc1)S(=O)(=O)c2cc(F)c([O-])c3ncccc23	6.31
6	Oc1ccc(c2cccnc12)S(=O)(=O)N3CCCC3	7.943
7	Oc1c(Cl)cc(c2cccnc12)S(=O)(=O)N3CCCC3	10
8	[O-]C1=CN2CCN(Cc3ccc(Cl)cc3Cl)CC2=CC1=O	10
9	[O-]C1=CN2CCN(Cc3c(Cl)cccc3Cl)CC2=CC1=O	10
10	Cc1ccc(C[N@@H+]2CCN3C=C([O-])C(=O)C=C3C2)c(C)c1	13
11	[O-]C1=CN2CCN(Cc3c(F)cccc3Cl)CC2=CC1=O	16
12	Oc1c(Cl)cc(c2cccnc12)S(=O)(=O)C3CCCC3	19.95
13	Cc1cc(c2cccnc2c1O)S(=O)(=O)N3CCCC3	19.95
14	Cc1ccc(cc1)S(=O)(=O)c2ccc(O)c3ncccc23	19.95
15	Oc1cc(\C=C(/C#N)\C(=O)N2CCC2)cc(c1[O-])[N+](=O)[O-]	33
16	[O-]C1=CN2CCN(Cc3c(F)cccc3F)CC2=CC1=O	38
17	[O-]C1=CN(C=CC1=O)c2cc(cc[nH+]2)c3ccccc3	40
18	[O-]C1=CN2CCN(Cc3ccc(F)cc3Cl)CC2=CC1=O	40
19	[O-]C1=CN2CCN(Cc3ccccc3Cl)CC2=CC1=O	40
20	Cc1cccc(CN2CCN3C=C([O-])C(=O)C=C3C2)c1C	40
21	[O-]C1=CN(C=CC1=O)c2cccc(c2)c3ccccc3	47
22	[O-]C1=CN2CCN(Cc3ccccc3F)CC2=CC1=O	50
23	Cc1ccccc1CN2CCN3C=C([O-])C(=O)C=C3C2	50
24	CCS(=O)(=O)c1ccc(O)c2ncccc12	50.12
25	CC1=CC(=O)C(=CN1c2cccc(c2)c3ccccc3)O	53
26	[O-]C1=CN(C=CC1=O)c2nccc(c2F)c3ccccc3	55

27	C[C@@H](O)C1=CC(=O)C(=CN1c2cccc(c2)c3ccccc3)O	61
28	Cc1ccc(C)c(CN2CCN3C=C([O-])C(=O)C=C3C2)c1	63
29	COc1ccc(cc1)C2(CC2)c3cc([nH]n3)c4sc(C)nc4C	75
30	[O-]C1=CN2CCN(Cc3ccccc3C#N)CC2=CC1=O	100
31	Oc1cc(\C=C(/C#N)\c2cnccn2)cc(c1O)[N+](=O)[O-]	108
32	[O-]C1=CN(C=CC1=O)c2cccc(n2)c3ccccc3	128
33	OCC1=CC(=O)C(=CN1c2cccc(c2)c3ccccc3)O	140
34	OCC1=CC(=O)C(=CN1c2ccc(cc2)c3ccccc3)O	140
35	CN1C(=O)C(=CN=C1c2cccc(c2)c3ccccc3)O	170
36	CCN1C(=O)S\C(=C\c2cc(O)c([O-])c(c2)[N+](=O)[O-])\C1=O	179
37	OB(O)C1=CN(C=C([O-])C1=O)c2cccc(c2)c3ccccc3	200
38	OC1=CN=C([N-]C1=O)c2ccc(cc2)C(F)(F)F	210
39	CCN(CC)C(=O)\C(=C(/N)\c1cc([O-])c(O)c(c1)[N+](=O)[O-])\C#N	214
40	[O-]C1=CN2CCN(Cc3ccccc3)CC2=CC1=O	220
41	COc1ccccc1CN2CCN3C=C([O-])C(=O)C=C3C2	250
42	Cc1ccc(cc1)c2ccc(O)c3ncccc23	251.19
43	COc1ccc(CN2CCN3C=C([O-])C(=O)C=C3C2)cc1	400
44	[O-]C1=CN2CC[N@H+](Cc3ccc(cc3)C(F)(F)F)CC2=CC1=O	450
45	OC1=CN(C=CC1=O)c2cc[nH+]c(c2)c3ccccc3	456
46	COc1ccc(cc1)[C@@H](C)c2cc([nH]n2)c3sc(C)nc3C	470
47	CCN(CC)C(=O)\C(=C(/[O-])\c1cc([O-])c(O)c(c1)[N+](=O)[O-])\C#N	551
48	[O-]C1=CN(C=CC1=O)c2cc(ccn2)c3cccnc3	574
49	OC1=CN=C([N-]C1=O)c2cccc(c2)C#Cc3ccccc3	590
50	[O-]C1=CN(C=CC1=O)c2cc(ccn2)c3ccncc3	672
51	Cc1cc(C)cc(c1)N2C=C(O)C(=O)C=C2CO	680
52	C[C@H](c1ccccc1)c2cc([nH]n2)c3sc(C)nc3C	700
53	[O-]C1=CN(C=CC1=O)c2cc(ccn2)c3ccccn3	826
54	OC1=CN=C([N-]C1=O)c2cccs2	860
55	COc1ccc(cc1)[C@H](C)c2cc([nH]n2)c3sc(C)nc3C	860
56	C[C@H](c1ccccc1)c2cc([nH]n2)c3sc(C)nc3C	900
57	[O-]C1=CN(C=CC1=O)c2nccc3ccccc23	962
58	COc1ccc(Cc2cc([nH]n2)c3sc(C)nc3C)cc1	1,000
59	C[C@@H](c1ccccc1)c2cc([nH]n2)c3sc(C)nc3C	1,100
60	Cc1nc(C)c(s1)c2cc([nH]n2)C(C)(C)c3ccccc3	1,100
61	Cc1ccc(cc1)S(=O)(=O)c2cc(O)c3ncccc3c2	1,584.89
62	CC(C)C[N@H+]1CCN2C=C([O-])C(=O)C=C2C1	1,600
63	Cc1cn2nc(ccc2n1)N3C=CC(=O)C(=C3)[O-]	1,667
64	Cc1ccc(cc1)N2C=C(O)C(=O)C=C2CO	1,700
65	[O-]C1=CN2CC[N@H+](Cc3ccc(cc3)C#N)CC2=CC1=O	2,000
66	Cc1nc(C)c(s1)c2cc(Cc3ccccc3)[nH]n2	2,000
67	[O-]C1=CN2CCN(CCc3ccccc3)CC2=CC1=O	2,000
68	COc1ccc(cc1)C2=C(N(C)C(=O)C(=C2)O)c3ccncc3	2,100
69	Cc1ccc(cc1)C(=O)c2ccc(O)c3ncccc23	3,981.07
70	OCC1=CC(=O)C(=CN1CCCc2ccccc2)[O-]	4,500
71	CC1=C(O)C(=O)C=CN1c2cc(C)cc(C)c2	5,500
72	Cc1ccc(N2C=C(O)C(=O)C=C2CO)c(C)c1	5,800
73	[O-]C1=CN2CCN(Cc3cccnc3)CC2=CC1=O	6,300
74	Cc1nc(C)c(s1)c2cc(CCc3ccccc3)[nH]n2	8,000
75	Cc1ccc(cc1)c2nc(C)c(s2)c3cc[nH]n3	11,000
76	Cc1nc(C)c(s1)c2cc[nH]n2	16,000
77	CC1=C(O)C(=O)C=CN1c2ccccc2	18,000
78	CCc1cc([nH]n1)c2sc(C)nc2C	20,000
79	CCc1nc(C)c(s1)c2cc[nH]n2	21,000

80	CC[C@H](c1ccccc1)c2cc([nH+][nH]2)c3sc(C)nc3C	21,000
81	Cc1nc(sc1c2cc[nH]n2)c3ccccc3	24,000
82	CCn1cc(c(C)n1)c2cc[nH]n2	69,000
83	Cc1ccn2c(c(C)[nH+]c2c1)c3cc[nH]n3	85,000
84	Cc1nc(C)c(s1)c2cc([nH]n2)c3ccccc3	130,000
Test Set		
1	[O-]c1c(F)cc(c2cccnc12)S(=O)(=O)c3ccc(F)cc3	3.981
2	[O-]C1=CN2CCN(Cc3ccccc3C(F)(F)F)CC2=CC1=O	60
3	CCN(CC)C(=O)\C(=C\c1cc(O)c([O-])c(c1)[N+](=O)[O-])\C#N	60
4	COc1cccc(\C=C\C(=O)c2cc(O)c(O)c(c2)[N+](=O)[O-])c1	70
5	COc1ccc(Oc2cccc(c2)C3=NC=C(O)C(=O)N3C)cc1	93
6	Cc1ccc(cc1)C(=O)c2cc(O)c(O)c(c2)[N+](=O)[O-]	127
7	CCCCc1ccc(cc1)N2C=C(O)C(=O)C=C2CO	160
8	CN1C(=O)C(=CN=C1c2ccc(Cl)c(c2)C(F)(F)F)O	180
9	Cc1nc(C)c(s1)c2cc([nH]n2)C3(CC3)c4ccccc4	210
10	[O-]C1=CN2C(=CC1=O)CSc3ccccc23	400
11	CC(C)C1=CC(=O)C(=CN1c2cccc(c2)c3ccccc3)O	410
12	CCC(CC)C[N@H+]1CCN2C=C([O-])C(=O)C=C2C1	630
13	[O-]C1=CN(C=CC1=O)c2ccnc([nH+]2)c3ccccc3	706
14	Oc1cccc2cccnc12	1,584.89
15	[O-]C1=CN(C=CC1=O)c2ccccc2	2,400
16	OCC1=CC(=O)C(=CN1c2ccccc2)O	3,400
17	Oc1c([O-])cc(cc1[N+](=O)[O-])\C(=C(/C#N)\C(=O)N2CCC2)\[O-]	3,560
18	Oc1ccc(c2cccnc12)S(=O)(=O)[O-]	6,309.57
19	[NH3+]CCC1=CC(=O)C(=CN1c2cccc(c2)c3ccccc3)O	13,000
20	Cc1nc(C)c(s1)c2cc(CCCc3ccccc3)[nH][nH+]2	34,000
21	Cc1ccc2[nH+]c(C)c(c3cc[nH]n3)n2c1	80,000

Table S6. 3D QSAR dataset of known A_{2A}AR inhibitors.

Ligand No.	Canonical SMILES	IC_{50} (nM)
Training Set		
1	Nc1nc(c2ccc(F)cc2)c(c3ccc(O)nc3)c4nc(Cc5ccc(F)cn5)nn14	0.3
2	Nc1nc(NCc2ccsc2)nc3sc([nH+]c13)c4occc4	0.36
3	CN1C=C(C=CC1=O)c2c(nc(N)n3nc(nc23)[C@H](O)c4ncccc4F)c5ccccc5	0.52
4	Nc1nc(NCc2ccccn2)nc3sc([nH+]c13)c4occc4	0.68
5	Nc1nc(NCCc2occc2)nc3sc([nH+]c13)c4occc4	2.93
6	COc1ccccc1CNc2nc(N)c3nc(sc3n2)c4ccccc4	5.34
7	COc1ccccc1Cn2cc3nc(nc(N)c3n2)c4occc4	6.21
8	COc1ccc(Cc2nc3c(C4=CN(C)C(=O)C=C4)c(nc(N)n3n2)c5ccccc5)nc1	7.72
9	Nc1nc(cn2c3ccccc3nc12)c4cccc(Br)c4	10.1
10	COc1cccc(c1)c2cn3c4ccccc4nc3c(N)n2	10.1
11	Cc1oc(cc1)c2nc(N)c3nn(Cc4ccccc4)cc3n2	10.91
12	Cc1oc(cc1)c2nc(N)nc3N(CCN4CCN(CC4)c5ccccc5F)C(=O)C=Nc23	11.7
13	Nc1nc2ccc(Cl)cc2c3[nH+]c(nn13)c4occc4	12
14	CC(=O)Nc1cc(nc(n1)n2nc(C)cc2C)c3cccc(n3)N4CCOCC4	13
15	Nc1nc2c(cnn2CCc3ccc(O)cc3)c4nc(nn14)c5occc5	14
16	Nc1nc(NCc2ccncc2)nc3sc([nH+]c13)c4occc4	15.3
17	COc1cc(OC)cc(c1)c2cc(NC(=O)C)nc(n2)n3nc(C)cc3C	17
18	COC1CN(C1)c2cccc(n2)c3cc(NC(=O)C)nc(n3)n4nc(C)cc4C	18
19	CCCCc1nc2[nH]c[nH+]c2c3nc(nn13)c4ccccc4Cl	18
20	COc1cccc(c1)c2cn3c4cc(F)c(F)cc4nc3c(N)n2	27

21	CCC(=O)Nc1cc(nc([nH+]1)n2nc(C)cc2C)c3cncc(OC)c3	31
22	Nc1nc(cn2c3ccccc3nc12)c4occc4	32.6
23	Nc1nc(cn2c3ccccc3nc12)c4ccc(F)cc4F	33.7
24	Nc1nc(cn2c3ccccc3nc12)c4ccc(Cl)cc4	35.4
25	Nc1nc(cn2c3ccccc3nc12)c4ccc(Br)cc4	38.4
26	Nc1nc(cn2c3ccccc3nc12)c4ccccc4	46.5
27	CCCCc1nc2[nH]c[nH+]c2c3nc(Cc4ccccc4)nn13	47
28	COc1cccc(CNc2nc(N)c3nc(sc3n2)c4ccccc4)c1	48
29	CCNc1c2c(nc(N)n3nc(nc23)c4occc4)nn1C	48
30	Cc1oc(cc1)c2nc(N)nc3N(CCN4CCN(CC4)c5ccccc5)C(=O)C=Nc23	49.2
31	COc1ccc(cc1)c2cn3c4ccccc4nc3c(N)n2	51.2
32	CCCCc1nc2[nH]c[nH+]c2c3nc(nn13)c4cccc(Cl)c4	56
33	CCCCc1nc2[nH]c[nH+]c2c3nc(nn13)c4ccc(O)cc4	58
34	Cc1oc(cc1)c2nc(N)nc3N(CCN4CCN(CC4)c5ccc(F)cc5)C(=O)C=Nc23	58.8
35	Clc1cc2nc(NCc3cccnc3)nc(C(=O)c4cccs4)c2s1	62
36	Nc1cc(c2occc2)c3oc(nc3c1)c4occc4	70.6
37	CCCCc1nc2[nH]c[nH+]c2c3nc(nn13)c4ccccc4	71
38	COc1ccccc1c2cn3c4ccccc4nc3c(N)n2	83.1
39	CCOc1ccc(cc1)C2=CN3C(=O)N(N=C3C(=N2)N)c4ccccc4	98
40	C[C@@H](Nc1nc(C(=O)c2ccc(C)s2)c3sccc3n1)c4cccnc4	99
41	Cc1c(nc(N)c2nc3ccccc3n12)c4ccccc4	113.7
42	Cc1ccc(s1)C(=O)c2nc(NCc3ccncn3)nc4ccsc24	116
43	C[C@@H](Nc1nc(C(=O)c2ccc(C)s2)c3sccc3n1)c4ccccn4	118
44	COc1ccc(CNc2nc(N)c3[nH+]c(sc3n2)c4occc4)cc1	123
45	CCNc1nc(C(=O)c2ccc(C)s2)c3sccc3n1	126
46	CCCCc1nc2[nH]ncc2c3nc(nn13)c4ccccc4Cl	130
47	Nc1nc(nc2sc(nc12)c3ccccc3)c4occc4	163
48	COC(=O)Nc1cc(nc(n1)n2nc(C)cc2C)c3cncc(OC)c3	167
49	CCCCc1nc2[nH]c[nH+]c2c3nc(nn13)c4ccc(C)cc4	180
50	Cc1ccc(s1)C(=O)c2nc(NCc3ccccn3)nc4ccsc24	186
51	CCCCc1nc2[nH]c[nH+]c2c3nc(nn13)c4cccc(C)c4	188
52	CCCCCc1nc2[nH]c[nH+]c2c3nc(nn13)c4ccccc4	200
53	Cc1oc(cc1)c2nc(N)nc3N(CCc4ccccc4)C(=O)C=Nc23	208
54	CCCCc1nc2[nH]c[nH+]c2c3nc(nn13)c4occc4	210
55	Nc1cc(NC(=O)CN2CCCCC2)cc3nc(oc13)c4occc4	250
56	C[C@H](O)CCc1nc(N)c2nc(n(C)c2n1)n3nccn3	260
57	CNc1cc2nc(NCc3cccnc3)nc(C(=O)c4cccs4)c2s1	276.8
58	Nc1nc(nc2sc(nc12)c3occc3)c4occc4	301
59	CN(C)c1cc2nc(NCc3cccnc3)nc(C(=O)c4cccs4)c2s1	318.3
60	Cc1ccc(s1)C(=O)c2nc(NCCc3ccccn3)nc4ccsc24	351
61	CCc1nc2[nH]c[nH+]c2c3nc(nn13)c4ccccc4	360
62	O=C(c1ccsc1)c2nc(NCc3cccnc3)nc4ccsc24	405
63	[O-]C(=O)C[C@@H]1CC[C@@H](CC1)Oc2ccc(cn2)c3ccc(cn3)c4nc5cc(ccc5[nH]4)C#N	430
64	Cc1ccc(cc1)C(=O)c2nc(NCc3cccnc3)nc4ccsc24	442
65	C[C@H](O)CCc1nc(N)c2nc(n(C)c2n1)n3nccn3	450
66	C[C@H](Nc1nc(C(=O)N2CCCC2)c3sccc3n1)c4cccnc4	468
67	Cc1oc(cc1)c2nc(N)nc3N(Cc4ccccc4)C(=O)C=Nc23	481.7
68	CCCCc1nc2[nH]c[nH+]c2c3nc(nn13)c4ccc(F)cc4	510
69	Cc1oc(cc1)c2nc(N)nc3N(CCCc4ccccc4)C(=O)C=Nc23	712
70	O=C(c1nccs1)c2nc(NCc3cccnc3)nc4ccsc24	732
71	CCCCc1nc2[nH]ncc2c3nc(nn13)c4cccc(Cl)c4	760
72	[O-]C(=O)CC1CCC(CC1)OC2CCN(CC2)c3ccc(cn3)c4nc5c(F)cccc5[nH]4	910
73	Cc1ccc(s1)C(=O)c2nc(NCc3cccnc3)nc4ccsc24	990

74	Cc1oc(cc1)c2nc(N)nc3N(CC4CC4)C(=O)C=Nc23	1,010
75	COc1ccccc1CN2C(=O)C=Nc3c2nc(N)nc3c4oc(C)cc4	1,016
76	Cc1oc(cc1)c2nc(N)nc3c2nc(O)n3Cc4ccccc4	1,030
77	O=C(c1cccnc1)c2nc(NCc3cccnc3)nc4ccsc24	1,168
78	Cc1oc(cc1)C(=O)c2nc(NCc3cccnc3)nc4ccsc24	1,252
79	COC(=O)Nc1nc2cc(ccc2[nH]1)S(=O)c3ccccc3	1,504
80	Clc1ccc(s1)C(=O)c2nc(NCc3cccnc3)nc4ccsc24	1,541
81	CCCCc1nc2[nH]c[nH+]c2c3[nH+]c(nn13)c4ccc(OC)cc4	1,600
82	O=C(c1ccccc1)c2nc(NCc3cccnc3)nc4ccsc24	1,619
83	Cc1[nH]c2nc(nc(NCc3ccncc3)c2c1C)c4ccccc4	1,711
84	O=C(c1ccncc1)c2nc(NCc3cccnc3)nc4ccsc24	1,942
85	Cn1c(nc2c(N)nc(CCCCO)nc12)n3nccn3	2,100
86	C[C@@H](Nc1nc(C(=O)N2CCCC2)c3sccc3n1)c4cccnc4	2,347
87	CCCCc1nc2[nH]ncc2c3[nH+]c(nn13)c4ccc(OC)cc4	2,600
88	CC(C)NC(=O)c1nc(NCc2cccnc2)nc3ccsc13	2,610
89	CCc1cc2nc(NCc3cccnc3)nc(C(=O)c4cccs4)c2s1	3,750
90	COc1ccccc1N2CCN(CCN3C(=O)C=Nc4c3nc(N)nc4c5oc(C)cc5)CC2	3,810.50
91	CCCCc1nc2ccccc2c3[nH+]c(nn13)c4ccc(O)cc4	5,300
92	O=C(N1CCCC1)c2nc(NCc3cccnc3)nc4ccsc24	6,136
93	Cc1ccc(s1)C(=O)c2ncnc3ccsc23	6,685
94	CNC(=O)c1nc(NCc2cccnc2)nc3ccsc13	7,107
95	CCCC(=O)Oc1ccc(CCNc2nc(N)c3nc(n(C)c3n2)n4nccn4)cc1	9,770
96	CNC(=O)[C@H]1O[C@H]([C@H](O)[C@@H]1O)n2cnc3c(NCCCCc4ccccc4)ncnc23	14,600
Test Set		
1	Nc1nc2c(cnn2CCc3ccccc3)c4nc(nn14)c5occc5	12
2	Nc1nc(SCc2cccc(CO)c2)c(C#N)c(c3occc3)c1C#N	22
3	CC(=O)Nc1cc(nc(n1)n2nc(C)cc2C)c3cccc(n3)N4CCC(O)CC4	22
4	CC(C)(C)c1ccc(cc1)c2cn3c4ccccc4nc3c(N)n2	33
5	CC(=O)Oc1ccc(CCNc2nc(N)n3nc(nc3n2)c4occc4)cc1	38.9
6	CCOC(=O)CCCOc1ccc(CCNc2nc(N)n3nc(nc3n2)c4occc4)cc1	50
7	COc1cncc(c1)c2cc(NC(=O)C)nc(n2)n3nc(C)cc3C	57
8	COCCN(C)c1cccc(n1)c2cc(NC(=O)C)nc(n2)n3nc(C)cc3C	70
9	Nc1nc(cn2c3ccccc3nc12)c4ccc(O)cc4	81.3
10	Cc1oc(cc1)c2nc(N)nc3N(CCC4CCN(CC4)c5ccccc5)C(=O)C=Nc23	279
11	C[C@@H](O)CCc1nc(N)c2nc(n(C)c2n1)n3nccn3	420
12	Cc1ccc2[nH]c(nc2n1)c3ccc(nc3)c4ccc(O[C@@H]5CC[C@H](CC(=O)[O-])CC5)nc4	490
13	Nc1nc2c(cnn2Cc3ccc(OCc4ccccc4)cc3)c5nc(nn15)c6occc6	500
14	Nc1nc(nc2nc(nn12)c3occc3)N4CCCN(CCc5cccc6NC(=O)Cc56)CC4	1,479
15	O=C(c1cncs1)c2nc(NCc3cccnc3)nc4ccsc24	1,715
16	Cc1[nH]c2nc(nc(N[C@@H]3CCCCNC3=O)c2c1C)c4ccccc4	2,060
17	CCNC(=O)c1nc(NCc2cccnc2)nc3ccsc13	2,516
18	CCCCc1nc2[nH]ncc2c3[nH+]c(nn13)c4ccc(OCC)cc4	2,700
19	CCOc1ccc(CCNc2nc(N)c(nc2Cl)C(=O)[NH+]=C(N)N)cc1	3,400
20	C=Cc1cc2nc(NCc3cccnc3)nc(C(=O)c4cccs4)c2s1	3,750
21	C[C@@H](Nc1nc(C(=O)N2CCCC2)c3sccc3n1)c4cccnc4	6,760
22	Cc1ccc(s1)C(=O)c2nc(C)nc3ccsc23	7,847
23	Clc1ccc(CO[C@H](Cn2ccnc2)c3ccc(Cl)cc3Cl)c(Cl)c1	14,253
24	CCNC(=O)[C@H]1O[C@H]([C@H](O)[C@@H]1O)n2cnc3c(NCCCCc4ccccc4)ncnc23	26,500

Table S7. Electronic properties of ligand forms of *R. palmatum* major metabolites (in eV).

Ligand	Form	ε_{HOMO}	ε_{LUMO}	$\Delta\varepsilon$	η	μ	ω
Aloe-emodin	1	-6.8537	-3.1767	3.6770	1.8385	-5.0152	6.8403
	2	-6.4134	-3.4512	2.9622	1.4811	-4.9323	8.2128
	3	-6.2245	-3.2879	2.9365	1.4683	-4.7562	7.7035
	4	-5.1081	-2.5442	2.5639	1.2820	-3.8261	5.7097
	5	-5.3011	-2.8360	2.4651	1.2326	-4.0686	6.7149
	6	-4.6440	-1.9205	2.7235	1.3617	-3.2822	3.9557
Chrysophanol	1	-6.8255	-3.1426	3.6829	1.8415	-4.9840	6.7448
	2	-6.3779	-3.3847	2.9933	1.4966	-4.8813	7.9602
	3	-6.2081	-3.2660	2.9421	1.4710	-4.7371	7.6271
	4	-5.0816	-2.4914	2.5902	1.2951	-3.7865	5.5353
	5	-5.0780	-2.8130	2.2650	1.1325	-3.9455	6.8729
	6	-4.5940	-1.8685	2.7255	1.3627	-3.2312	3.8308
Emodin	1	-5.1484	-2.5171	2.6313	1.3157	-3.8328	5.5829
	2	-5.1222	-2.8273	2.2949	1.1475	-3.9747	6.8842
	3	-4.9383	-2.8029	2.1354	1.0677	-3.8706	7.0159
	4	-4.6185	-1.8930	2.7255	1.3627	-3.2558	3.8892
	5	-4.6926	-2.3628	2.3298	1.1649	-3.5277	5.3416
Physcion	1	-6.7375	-3.1065	3.6310	1.8155	-4.9220	6.6719
	2	-6.1298	-3.2090	2.9208	1.4604	-4.6694	7.4648
	3	-6.3698	-3.4146	2.9551	1.4776	-4.8922	8.0989
	4	-5.1309	-2.4979	2.6329	1.3165	-3.8144	5.5260
	5	-5.2727	-2.7692	2.5034	1.2517	-4.0210	6.4584
	6	-4.6005	-1.8755	2.7250	1.3625	-3.2380	3.8475
Rhein	1	-6.1419	-3.1308	3.0111	1.5055	-4.6363	7.1387
	2	-6.1363	-3.3761	2.7601	1.3801	-4.7562	8.1958
	3	-6.1499	-3.2503	2.8997	1.4498	-4.7001	7.6185
	4	-4.9859	-2.4559	2.5300	1.2650	-3.7209	5.4723
	5	-5.1946	-2.7709	2.4237	1.2119	-3.9828	6.5447
	6	-4.5353	-1.8210	2.7143	1.3571	-3.1782	3.7213
Dopamine *		-6.2276	-0.4518	5.7758	2.8879	-5.0152	1.9310
Levodopa *		-6.2883	-0.5905	5.6979	2.8489	-4.9323	2.0761

* Standard drugs.

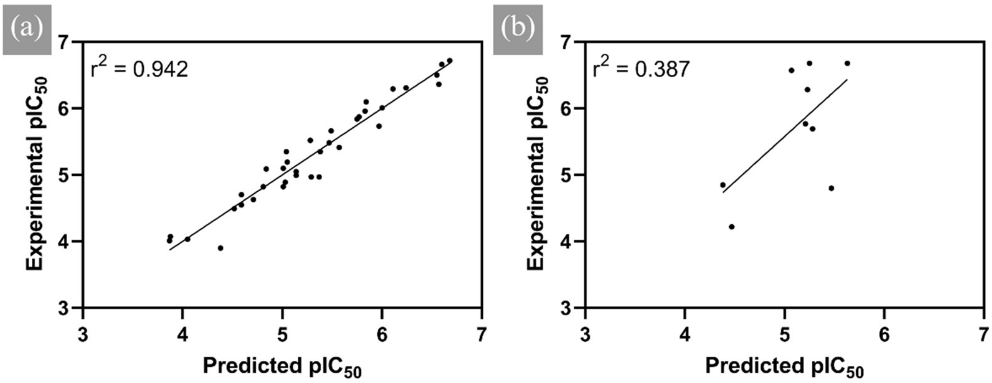

Figure S1. Experimental and predicted IC_{50} correlation of (a) training dataset and (b) test dataset for a 3D-QSAR model of ASN.

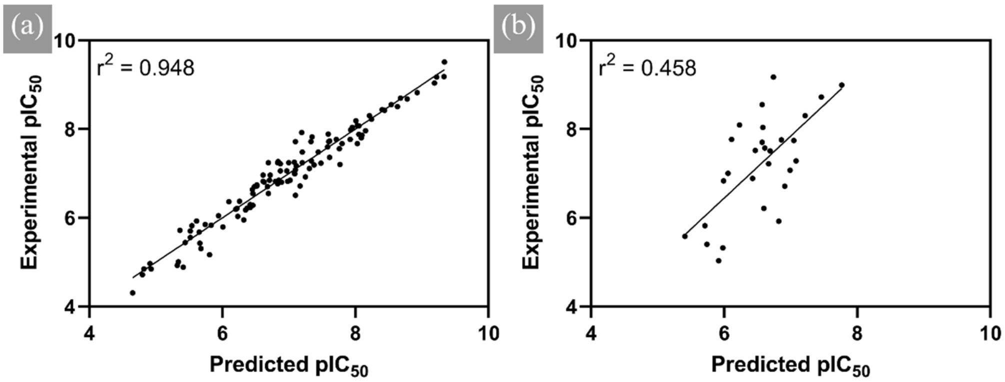

Figure S2. Experimental and predicted IC_{50} correlation of (a) training dataset and (b) test dataset for a 3D-QSAR model of MAOB.

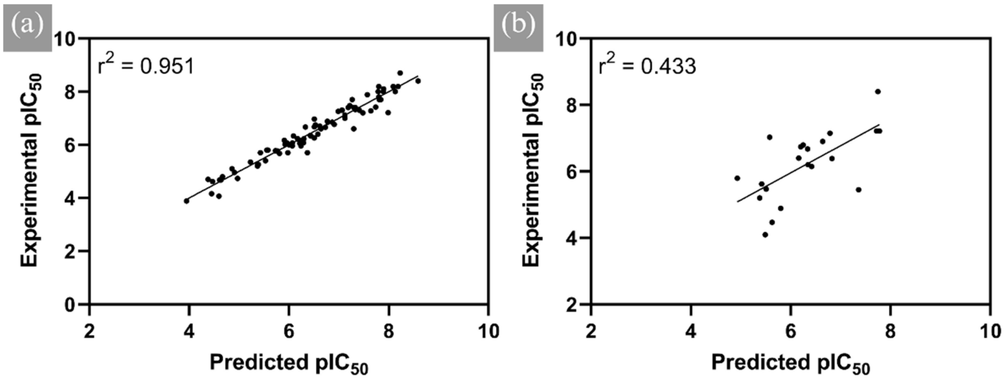

Figure S3. Experimental and predicted IC_{50} correlation of (a) training dataset and (b) test dataset for a 3D-QSAR model of COMT.

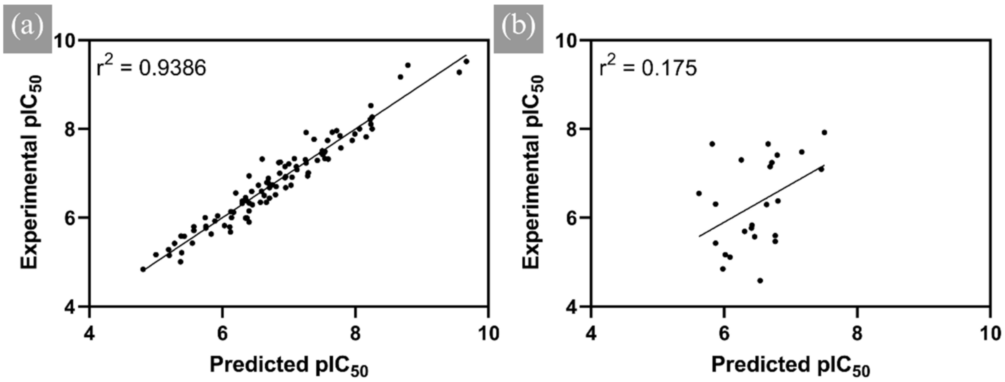

Figure S4. Experimental and predicted IC_{50} correlation of (a) training dataset and (b) test dataset for a 3D-QSAR model of $A_{2A}AR$.

References

1. Ulmer, T.S.; Bax, A.; Cole, N.B.; Nussbaum, R.L. Structure and Dynamics of Micelle-Bound Human α-Synuclein. *J. Biol. Chem.* **2005**, *280*, 9595–9603. https://doi.org/10.1074/jbc.M411805200.
2. Gnanaraj, C.; Sekar, M.; Fuloria, S.; Swain, S.S.; Gan, S.H.; Chidambaram, K.; Rani, N.N.I.M.; Balan, T.; Stephenie, S.; Lum, P.T.; et al. In Silico Molecular Docking Analysis of Karanjin against Alzheimer's and Parkinson's Diseases as a Potential Natural Lead Molecule for New Drug Design, Development and Therapy. *Molecules* **2022**, *27*, 2834. https://doi.org/10.3390/molecules27092834.
3. Hubálek, F.; Binda, C.; Li, M.; Herzig, Y.; Sterling, J.; Youdim, M.B.H.; Mattevi, A.; Edmondson, D.E. Inactivation of Purified Human Recombinant Monoamine Oxidases A and B by Rasagiline and Its Analogues. *J. Med. Chem.* **2004**, *47*, 1760–1766, doi:10.1021/jm0310885.
4. Binda, C.; Hubálek, F.; Li, M.; Herzig, Y.; Sterling, J.; Edmondson, D.E.; Mattevi, A. Binding of Rasagiline-Related Inhibitors to Human Monoamine Oxidases: A Kinetic and Crystallographic Analysis. *J. Med. Chem.* **2005**, *48*, 8148–8154, doi:10.1021/jm0506266.
5. Rutherford, K.; Le Trong, I.; Stenkamp, R.E.; Parson, W.W. Crystal Structures of Human 108V and 108M Catechol O-Methyltransferase. *J. Mol. Biol.* **2008**, *380*, 120–130. https://doi.org/10.1016/j.jmb.2008.04.040.
6. Jaakola, V.-P.; Griffith, M.T.; Hanson, M.A.; Cherezov, V.; Chien, E.Y.T.; Lane, J.R.; IJzerman, A.P.; Stevens, R.C. The 2.6 Angstrom Crystal Structure of a Human A_{2A} Adenosine Receptor Bound to an Antagonist. *Science* **2008**, *322*, 1211–1217. https://doi.org/10.1126/science.1164772.
